# Designing for implementation: a cognitive task analysis of intimate partner violence screening in hospital trauma care in Alberta, Canada

**DOI:** 10.3389/frhs.2026.1743548

**Published:** 2026-03-03

**Authors:** Stephanie Montesanti, Nori Bradley, Sarah Demedeiros, Rhyann McKay

**Affiliations:** 1 CARE Research Lab, School of Public Health, University of Alberta, Edmonton, AB, Canada; 2 Centre for Healthy Communities, School of Public Health, University of Alberta, Edmonton, AB, Canada; 3 Alberta Strategy for Patient-Oriented Research (SPOR) Learning Health System Team, Edmonton, AB, Canada; 4 University of Alberta Hospital, Edmonton, AB, Canada; 5 Department of Surgery, Faculty of Medicine & Dentistry, University of Alberta, Edmonton, AB, Canada

**Keywords:** clinical workflow integration, cognitive task analysis, complex adaptive systems, complexity science, implementation feasibility, implementation science, intimate partner violence, IPV screening

## Abstract

**Background:**

Intimate partner violence (IPV) has serious health consequences, yet routine IPV screening remains inconsistently implemented in hospital trauma centres. Despite evidence supporting screening, implementation challenges persist. This study used Cognitive Task Analysis (CTA) to examine how trauma care providers perceive and enact IPV screening, with attention to cognitive processes, barriers, and facilitators to implementation.

**Methods:**

We conducted CTA group interviews with nine trauma care providers from two trauma centers in Edmonton, Alberta, Canada. Participants included trauma surgeons, nurse practitioners, social workers, and patient care managers. Using a structured interview guide and concept mapping techniques, we elicited knowledge structures, decision-making processes, and perceived constraints related to IPV screening. We applied an interpretive qualitative approach to uncover underlying themes related to cognitive work and task complexity. Grounded theory techniques, such as open and axial coding, were used in conjunction with CTA to analyze how participants reasoned through clinical scenarios. We paid close attention to how providers assessed cues, coordinated across roles, shifted priorities, and navigated organizational constraints. This hybrid approach allowed us to bridge systems-level implementation science with cognitive insights, drawing conceptually on CFIR and Proctor et al.'s implementation outcomes to generate actionable knowledge for IPV screening interventions in trauma care settings.

**Results:**

Themes were synthesized into six overarching cognitive domains: trauma care workflow, team collaboration and knowledge, critical situations and decision-making, IPV screening practices and challenges, understanding patient experiences, and institutional support. These were further illustrated through refined concept maps that visually represented participants' mental models, task sequences, and decision-making strategies.

**Conclusion:**

Trauma care providers are well-positioned to identify IPV, yet screening is constrained by limited institutional support, unclear procedures, and poor integration into trauma workflows. Findings highlight the need for system-level strategies that align IPV screening with the cognitive and organizational realities of trauma care. By applying CTA, this study informs the design and implementation of context-sensitive IPV screening interventions that are more acceptable, appropriate, and feasible in hospital trauma settings. Furthermore, this study informs implementation strategies for integrating IPV screening interventions into trauma care, with particular implications for improving the acceptability, appropriateness, feasibility, and sustainability of evidence-based practices.

## Background

Intimate partner violence (IPV) is a pervasive public health issue affecting individuals across all demographics, with a disproportionate impact on women and gender-diverse populations (1, 2). IPV includes physical, sexual, or psychological harm inflicted by a current or former partner in a romantic or dating relationship. Globally, one in three women has experienced physical or sexual IPV during her lifetime (2).

**Table 1:** CTA process following crandall et al. (26) five steps.

	Steps	Process
1.	Collecting Knowledge Representations	We began by identifying the domain and scope of interest (IPV screening and assessment in trauma care) and selecting knowledgeable participants with diverse roles.
2.	Task Diagramming and Knowledge Audit	We conducted a knowledge audit using a structured set of interview questions (see Appendix A) to map the trauma care workflow and elicit the cognitive demands associated with patient care, task coordination, and IPV-related decision-making.
3.	Simulation Interviews and Probing for Expertise	Participants were asked to recall specific instances of treating trauma patients where IPV was suspected or disclosed. Using probes adapted from the CDM (e.g., “What cues were you looking for?”, “What knowledge guided your decision?”), we explored how they adapted routines, interpreted cues, and collaborated with colleagues.
4.	Concept Mapping	To visualize the knowledge structures, we worked with participants to co-create concept maps that represented their cognitive processes, workflows, and perceived barriers related to IPV screening and assessment.
5.	Data Interpretation and Model Construction	Interview transcripts and maps were analyzed to construct cognitive models of trauma care providers' reasoning and the barriers to implementing IPV screening interventions.

In Canada, IPV remains the leading cause of severe injuries and the second leading cause of death among women of reproductive age (41). Emerging research highlights that women experiencing IPV are at a high risk for injuries to the head, neck, and face that can lead to a traumatic brain injury (TBI) (3–5). Additionally, non-fatal strangulation may result in acquired brain injuries (ABIs) through hypoxia or anoxia—conditions caused by restricted or halted oxygen supply to the brain (5, 6).

Retrospective analyses indicate that nearly half of women murdered by an intimate partner had sought healthcare for IPV-related injuries within two years before their deaths, representing critical missed opportunities for early intervention in healthcare settings (7). The increased service use due to IPV-related injuries has also significantly burdened healthcare systems and led to higher costs (8).

Emergency departments (EDs) and trauma centres are often the initial points of contact for individuals with IPV-related injuries, positioning them as crucial sites for early identification and response. Studies indicate that up to 54% of women presenting at EDs have experienced IPV, yet only about 5% are detected by healthcare providers (9, 10). Professional bodies such as the American College of Surgeons and the Trauma Association of Canada recommend routine IPV screening for patients with traumatic injuries (11), but consistent implementation in clinical practice remains limited. When universal screening is conducted, studies show that 16% of women—and 11.3% of patients overall—admitted to trauma services report IPV-related injuries (12). Implementing effective IPV screening and disclosure in trauma care settings is hindered by systemic barriers such as a lack of privacy, time constraints, and limited access to support services. In Canada, these challenges are compounded by the absence of standardized screening protocols and clear institutional guidelines for identifying IPV in hospital settings (7). Furthermore, no single IPV screening program that integrates an appropriate screening tool, embedded clinician supports, referral protocol, and resources is recommended for Canadian trauma centres.

To examine how such challenges have been addressed elsewhere, our team conducted a rapid realist review (unpublished) to evaluate evidence on effective IPV screening programs for hospitalized injured patients in international acute care settings, including trauma care environments. Our inclusion criteria for the review encompassed articles describing the context and mechanisms for implementing comprehensive IPV screening programs, which include a screening tool (universal or case finding), provider education, referral supports, and other system-level supports (e.g., placing screening prompts or reminders), as well as program outcomes from evaluation studies. Out of 10 articles that met our inclusion criteria, only three studies described the implementation outcomes of comprehensive IPV screening programs that integrated strategies beyond just a screening tool—such as training, clear referral pathways, and embedded supports (e.g., email reminders or prompts, availability of social workers, hiring an advocate). This highlights the complexities of implementing multi-component interventions, which require more time, resources, and coordination across teams and systems.

The busy nature of acute trauma care—with its time pressures, high-acuity cases, interdisciplinary coordination, and rapid decision-making—poses additional challenges for integrating IPV screening into routine workflows. Existing research has primarily explored institutional and provider-level barriers, such as knowledge, confidence, and attitudes (13–16). However, these studies provide limited insight into how trauma care providers think, make decisions, and act in real-time when encountering potential IPV cases. As a result, a deeper understanding of the cognitive demands and decision-making processes that influence provider behaviour is essential for designing effective IPV screening programs, including decisions about when, how, and by whom screening should be carried out in trauma care settings.

The fields of implementation science (17, 18) and complexity science (19, 20) offer complementary approaches to understanding how IPV screening programs can be effectively integrated into the dynamic, real-world context of trauma care. Implementation science emphasizes tailoring interventions to local context, identifying barriers and facilitators, and engaging system actors in co-designing solutions that fit within existing workflows (18, 20). However, to date, few IPV screening studies have applied formal implementation frameworks or explored the cognitive aspects of provider decision-making and task performance, which are essential for guiding adoption and sustainment, especially in determining whether and how screening practices are integrated into busy clinical settings.

Complexity science contributes to this understanding by conceptualizing healthcare settings as interconnected, constantly evolving systems in which change occurs in unpredictable and emergent ways. In such settings, complexity science encourages close attention to how work unfolds in real-time, how clinicians respond to unexpected situations, and how small changes can produce significant system-wide effects (21). In trauma care, these dynamics are particularly evident, as clinicians often work under high pressure, in shifting teams, and with limited time and resources (22), requiring responsive, flexible, and adaptable practice. IPV screening is especially sensitive to these dynamics because it relies on trust, timing, and the ability to respond to each patient's unique situation (23).

Together, implementation science and complexity science lay a strong foundation for applying Cognitive Task Analysis (CTA)—a set of qualitative methods developed in cognitive psychology and human factors to uncover the tacit knowledge and decision strategies used by experts in high-stakes environments (24). CTA methods were created to understand decision-making in complex, uncertain, and challenging contexts (25). According to a systematic review by Swaby et al. (25), CTA has been widely used in surgery and intensive care research. However, it remains underutilized in studies related to the design and implementation of IPV programs.

In this study, we use CTA to explore how trauma care providers think and make decisions in real-time when encountering patients with potential IPV-related injuries. By integrating approaches from cognitive, implementation, and complexity sciences, we aim to inform IPV screening programs (i.e., incorporate multiple program components, including institutional support) that are contextually grounded, practically feasible, and responsive to the realities of acute care environments.

## Methods

### Research objectives

The following research objectives guided our CTA study:

To understand the cognitive aspects of the tasks involved in trauma care
To elicit key knowledge and understanding of IPV among trauma care providers
To identify aspects of trauma care providers' work that pose barriers to IPV screening among hospitalized trauma care patients
To co-develop concept maps to convey trauma care providers' knowledge on IPV screening practices within trauma service

Objectives 1 and 2 focused on understanding cognitive processes to inform the *design* of IPV screening programs, while objectives 3 and 4 focused on understanding the cognitive processes to guide the *implementation* of IPV screening programs.

### Study design

This study used a Cognitive Task Analysis (CTA) approach, following the methodological guidance outlined in *Working Minds: A Practitioner's Guide to Cognitive Task Analysis* by Crandall et al. (26). CTA involves a series of qualitative methods aimed at uncovering the knowledge, decision-making processes, and cognitive demands of individuals working in complex, high-stakes environments. Key attributes of the CTA methodology include: *time* (e.g., gathering information in the present as tasks are performed, reviewing past instances such as caring for a patient who experienced IPV, and considering future changes to tasks or performance needed to facilitate IPV screening and assessment); *realism* (e.g., focusing on the performance of real-world tasks); and *difficulty* (e.g., understanding tasks that may be highly routine and specialized). Given the cognitively intense nature of trauma care and the underutilization of IPV screening in these settings, CTA was deemed an appropriate methodology to explore the clinical reasoning that underpins trauma care providers' actions and judgments related to IPV identification and response. CTA methods are typically interview-based, recognizing that the voices of experts are necessary to elicit complex cognition at work (27).

### Setting and participants

The study was conducted at two urban trauma centres in Edmonton, Alberta, Canada: the University of Alberta Hospital (a Level 1 trauma centre) and the Royal Alexandra Hospital (a Level 2 trauma centre). Trauma centres are designated by levels that reflect their resources and capabilities for managing traumatic injuries—Level 1 being the highest and Level 3 the lowest. Level 1 centres provide comprehensive care, including 24/7 access to specialized medical and surgical services, advanced diagnostic technology, and multidisciplinary trauma teams. They also have mandated roles in research, education, and quality improvement. In contrast, Level 2 trauma centres offer similar acute care services but may lack some of the subspecialty and academic infrastructure of Level 1 centres. Lower-level trauma centres (e.g., Level 3) typically provide initial stabilization and coordinate transfer to higher-level facilities for definitive care.

Alberta has one of the highest rates of IPV in Canada (28), making it a relevant setting for exploring the integration of an IPV screening program into trauma care. We used purposive sampling to recruit a multidisciplinary sample of trauma care providers across various professional roles, including trauma surgeons, nurse practitioners, social workers, registered nurses, and a patient care manager. Eligibility criteria included current employment in trauma services and direct experience with the acute care management of injured patients.

A total of nine trauma care providers participated in the study. This sample size is consistent with CTA guidelines, which suggest a minimum of three to five experts is needed to identify expert cognitive processes (26, 29). Six participants were recruited from the University of Alberta Hospital and three from the Royal Alexandra Hospital. The sample included three trauma surgeons, two nurse practitioners, two social workers, one patient care manager, and one charge nurse. This composition allowed for a diverse exploration of cognitive processes and knowledge structures related to IPV screening interventions in trauma care.

### CTA procedure

Several methods of knowledge elicitation are described within CTA, as defined by Crandall et al. (26). Among these, we incorporated two complementary methods: the Critical Decision Method (CDM) and Applied Cognitive Task Analysis (ACTA) (30). We used CDM, a semi-structured, retrospective interview technique developed to elicit decision-making processes in high-stakes situations, to probe participants' experiences with IPV screening during critical or atypical trauma cases. This method allowed us to examine how providers recognized cues, assessed situations, adapted their routines, and made time-sensitive decisions under pressure. In parallel, ACTA interview techniques were used to capture more routine cognitive demands across the trauma care workflow. ACTA emphasizes task decomposition and knowledge auditing (i.e., identifying the types of knowledge experts rely on to perform a task), and was particularly useful for mapping provider roles, interprofessional coordination, and information flow during typical trauma care scenarios (31). Together, CDM and ACTA provided a more comprehensive understanding of the cognitive and contextual conditions that shape IPV screening and assessment behaviour in trauma care settings. Table 1 describes the five steps used in the CTA process.

### Data collection

Before conducting the CTA interviews, the research team participated in guided walkthroughs of the trauma units at the University of Alberta Hospital and the Royal Alexandra Hospital. These site visits provided contextual insight into the physical layout, workflow, and unit dynamics. Observational notes were captured to inform interview facilitation. Group CTA interviews were held virtually using Zoom™ conferencing software at each hospital site, facilitated by a researcher trained in CTA (SM). Each session began with an overview of the study objectives, followed by structured questions based on a CTA-informed interview guide. The group interviews lasted 90 min, were audio-recorded, and transcribed verbatim.

### Data analysis

**Figure 1:**
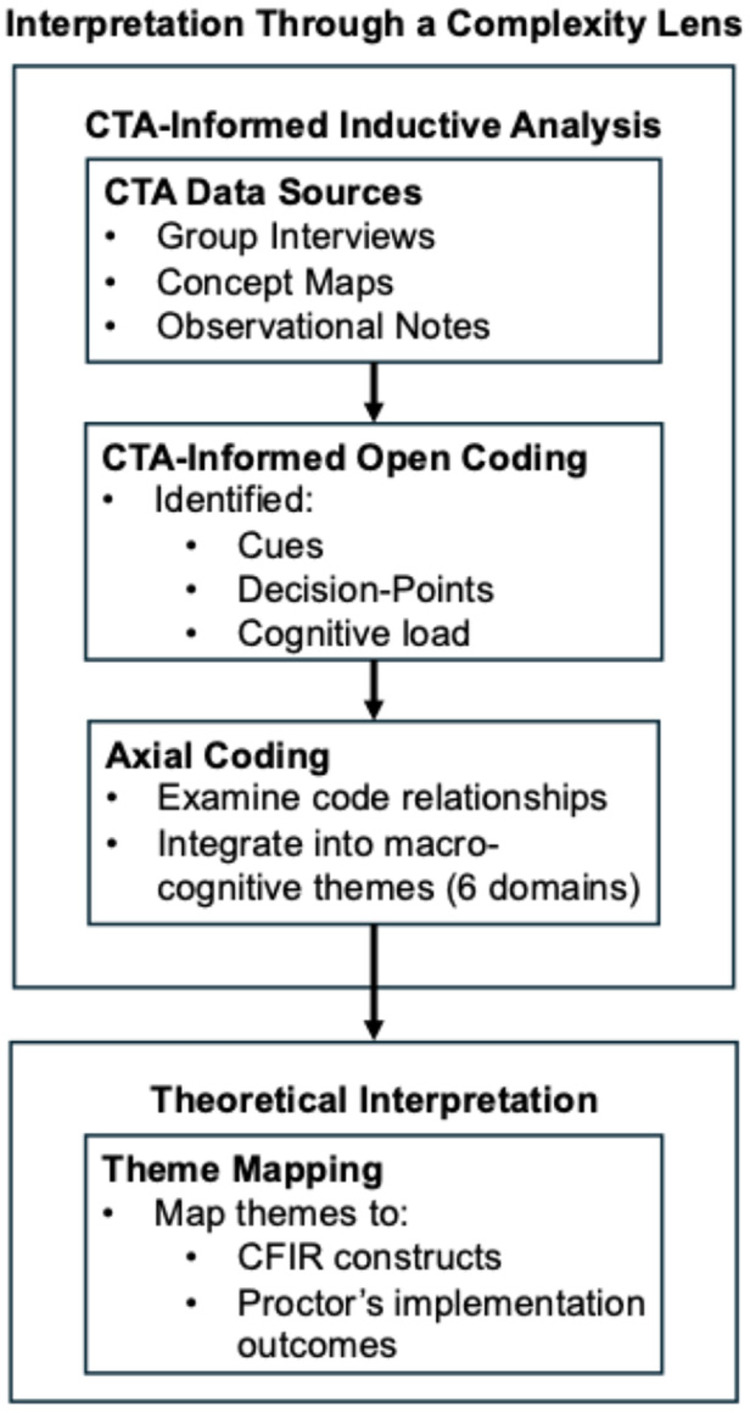
Analytic process. CTA group interview transcripts, concept maps, and observational notes were analyzed using CTA-informed open coding and grounded theory techniques. Codes were aggregated through axial coding into six macro-cognitive themes, which were then interpreted using CFIR and Proctor et al.'s implementation outcomes.

We employed a hybrid analytical approach that combined elements of grounded theory (32) with the structured procedures outlined by Crandall et al. (26). This approach enabled us to examine both the cognitive strategies and contextual factors influencing IPV screening in trauma care settings. This analysis was informed by principles of implementation science and complexity science, which provided conceptual lenses for interpreting how individual cognition and system-level conditions interact in sharing IPV screening practices. We drew conceptually from the Consolidated Framework for Implementation Research (CFIR) (33) and Proctor et al.'s (34) taxonomy of implementation outcomes to situate emerging themes within broader domains of implementation, such as intervention characteristics, organizational context, and individual provider factors (see Figure 1). This orientation supported our interpretation of findings in relation to key implementation constructs, including the acceptability, appropriateness, and feasibility of IPV screening interventions in complex, time-sensitive trauma care environments.

Data analysis proceeded in four iterative and overlapping phases: preparation, data structuring, discovering meaning, and representing findings (26). In the **preparation phase,** the research team (SM, SD and Research Assistant) listened to audio recordings and reviewed transcripts to become familiar with the interview data, and began visualizing concept maps with participants. This phase allowed us to focus the analysis on key task domains, cognitive strategies, and contextual factors related to IPV screening.

The **data structuring** phase involved open coding of transcripts to identify recurring patterns related to cognitive work, decision-making, and knowledge use. We employed a coding framework based on CTA constructs, including situational awareness, task goals, decision-making cues, mental models, and knowledge requirements (26), combined with grounded theory's constant comparative method, to stay open to emergent themes. Transcripts were independently coded by two team members (SM and a Research Assistant) and compared to ensure consistency and rigour.

In the **discovering meaning** phase, we examined the relationships among codes to understand how trauma care providers recognized cues, adapted workflows, coordinated with other providers, and made judgment calls about IPV screening. We explored both routine and atypical decision-making processes to uncover how tacit knowledge and contextual pressures influenced behaviour and decision-making. Through axial coding and iterative team discussion, we refined thematic categories and linked them to broader cognitive domains.

**Figure 2:**
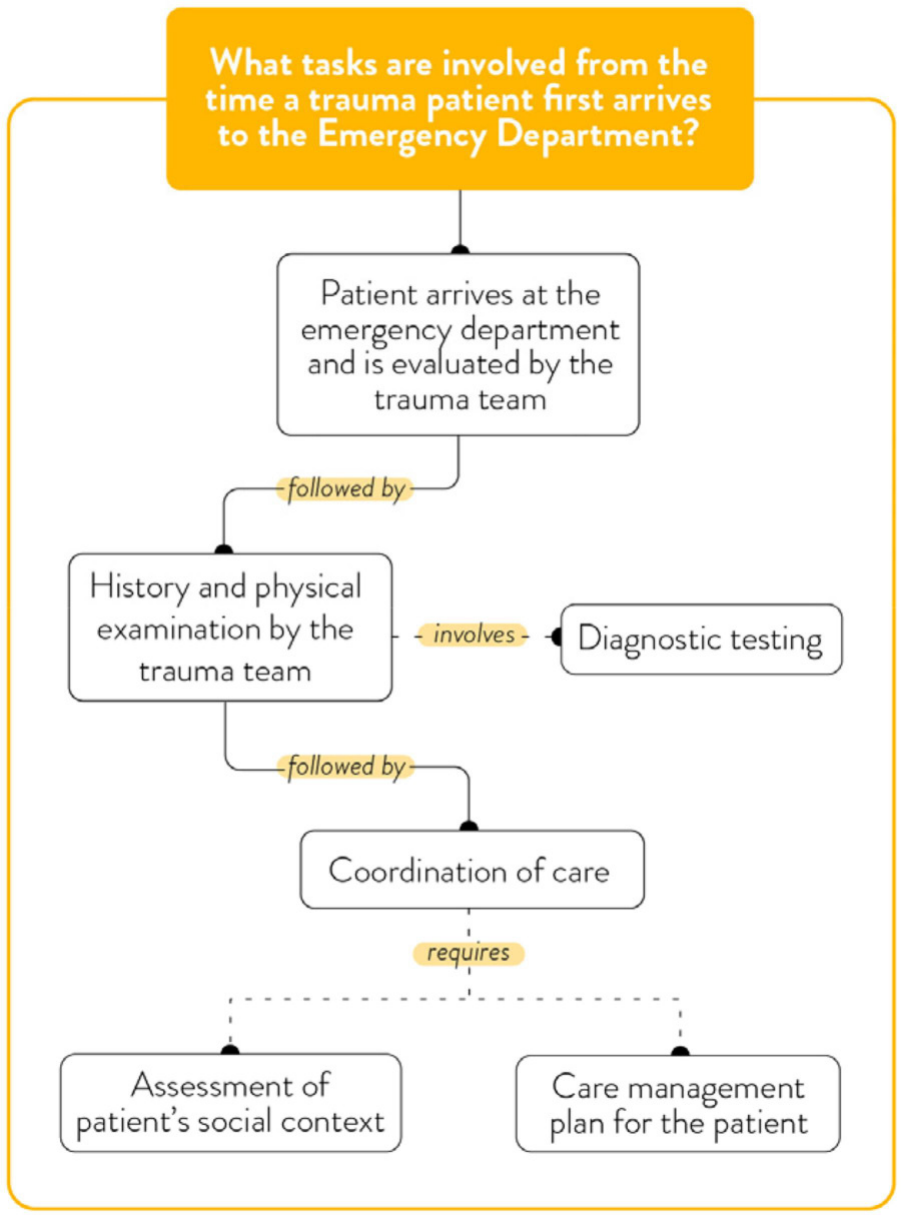
The trauma care workflow.

**Figure 3:**
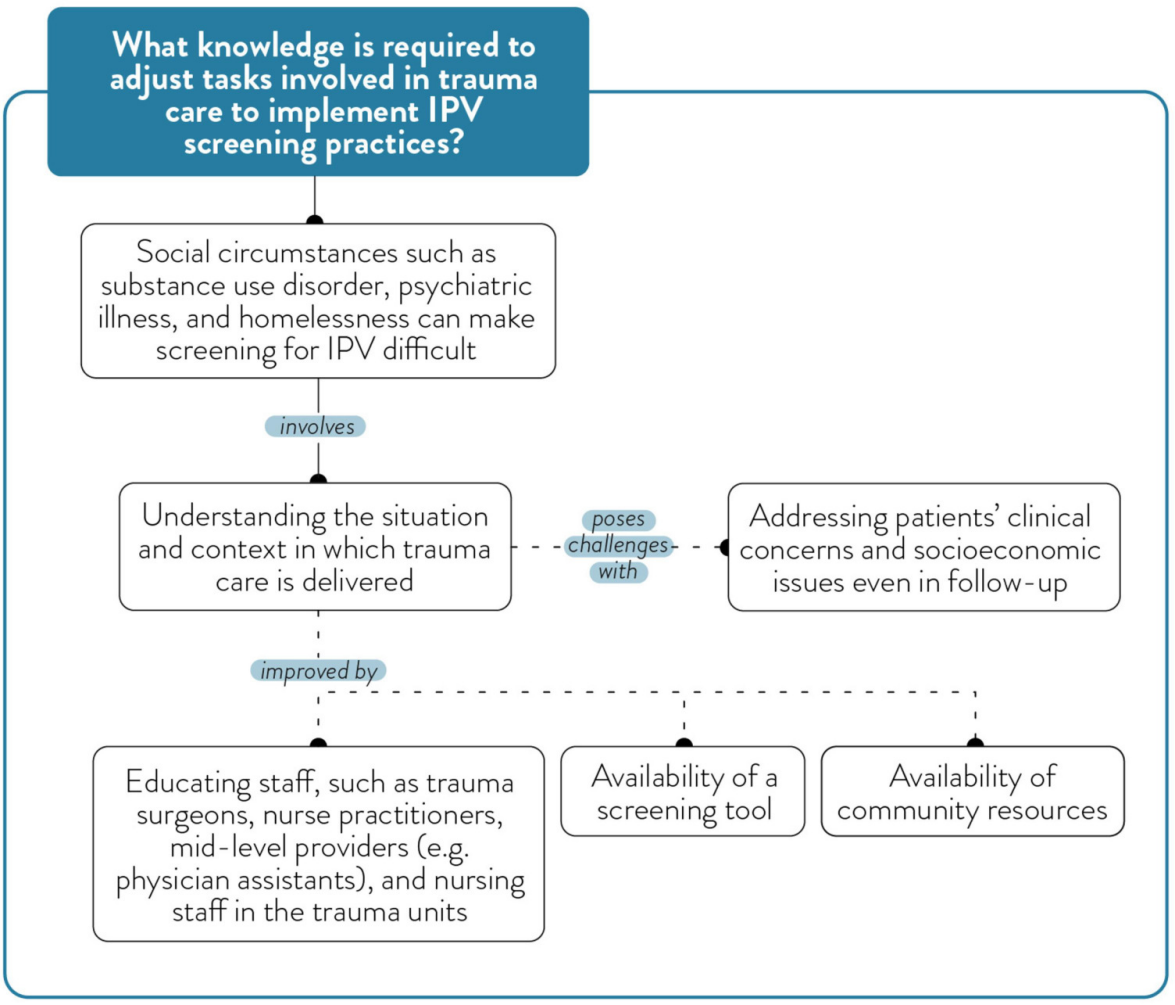
Team knowledge and collaboration.

**Figure 4:**
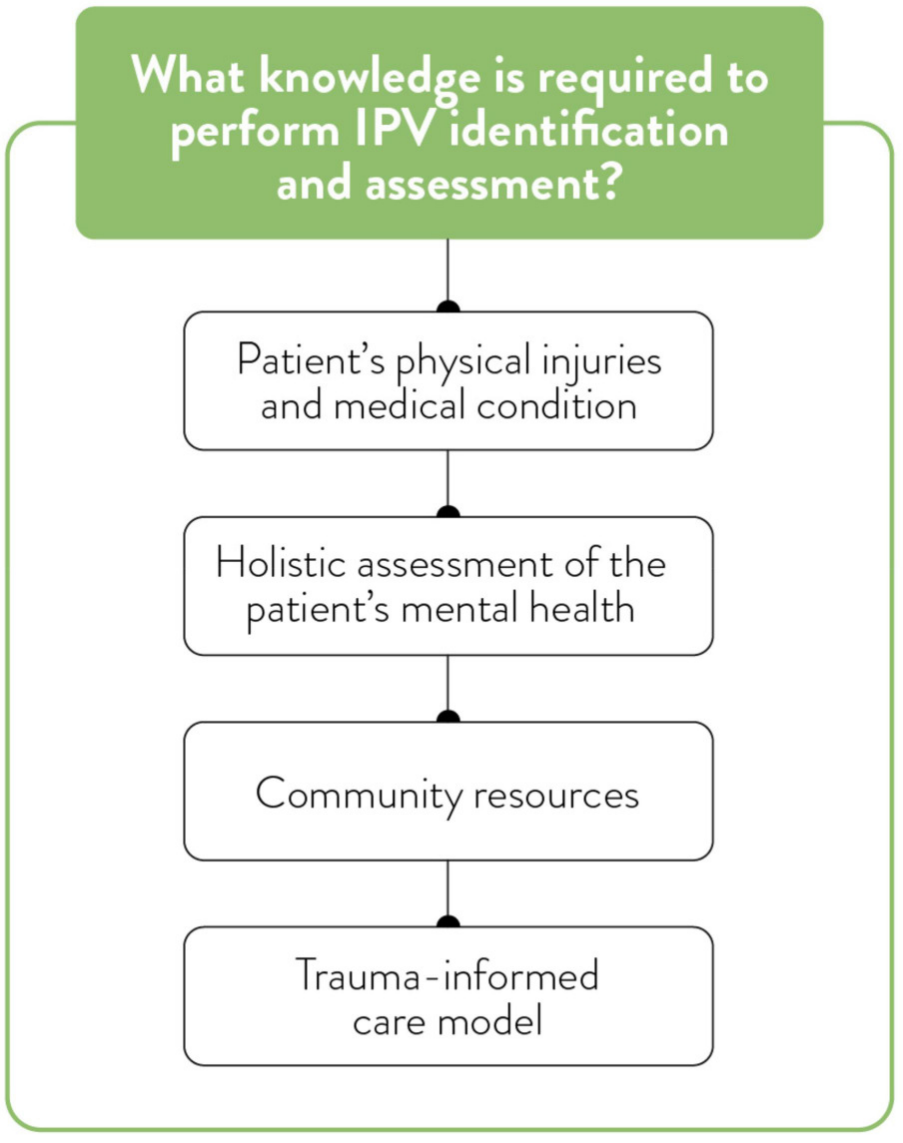
Knowledge to identify and assess IPV.

**Figure 5:**
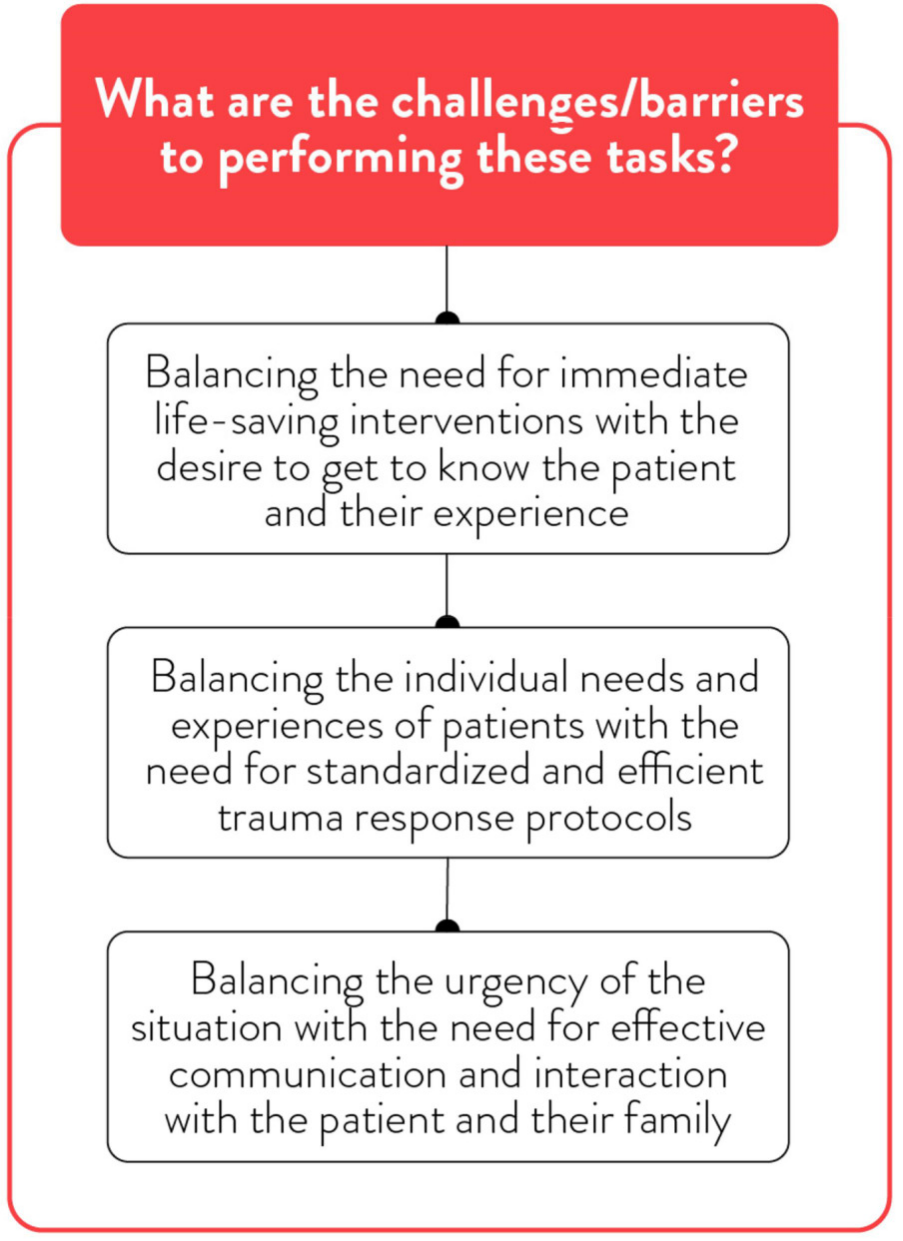
Challenges and barriers for IPV screening.

**Figure 6:**
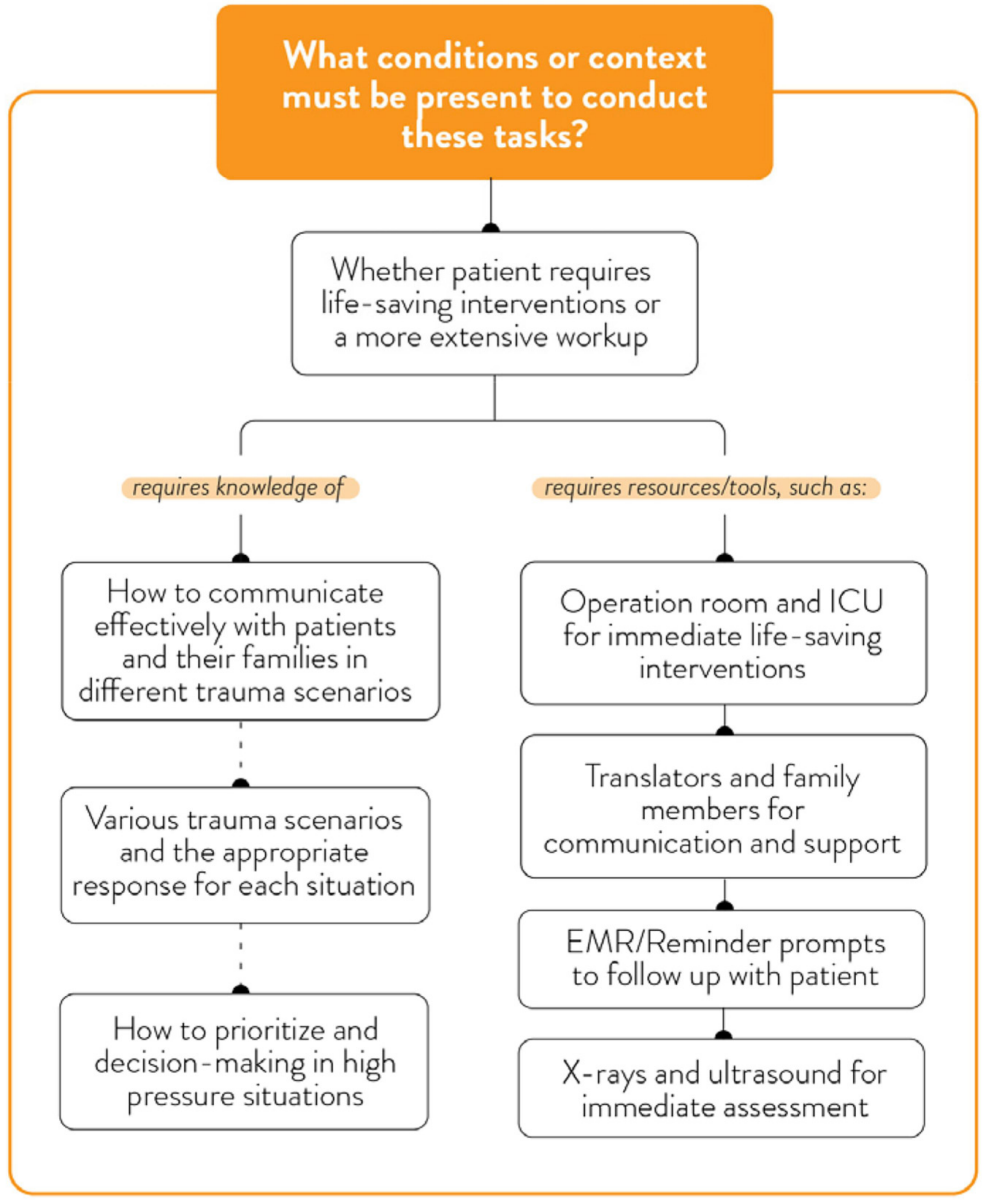
Understanding the patient.

**Figure 7:**
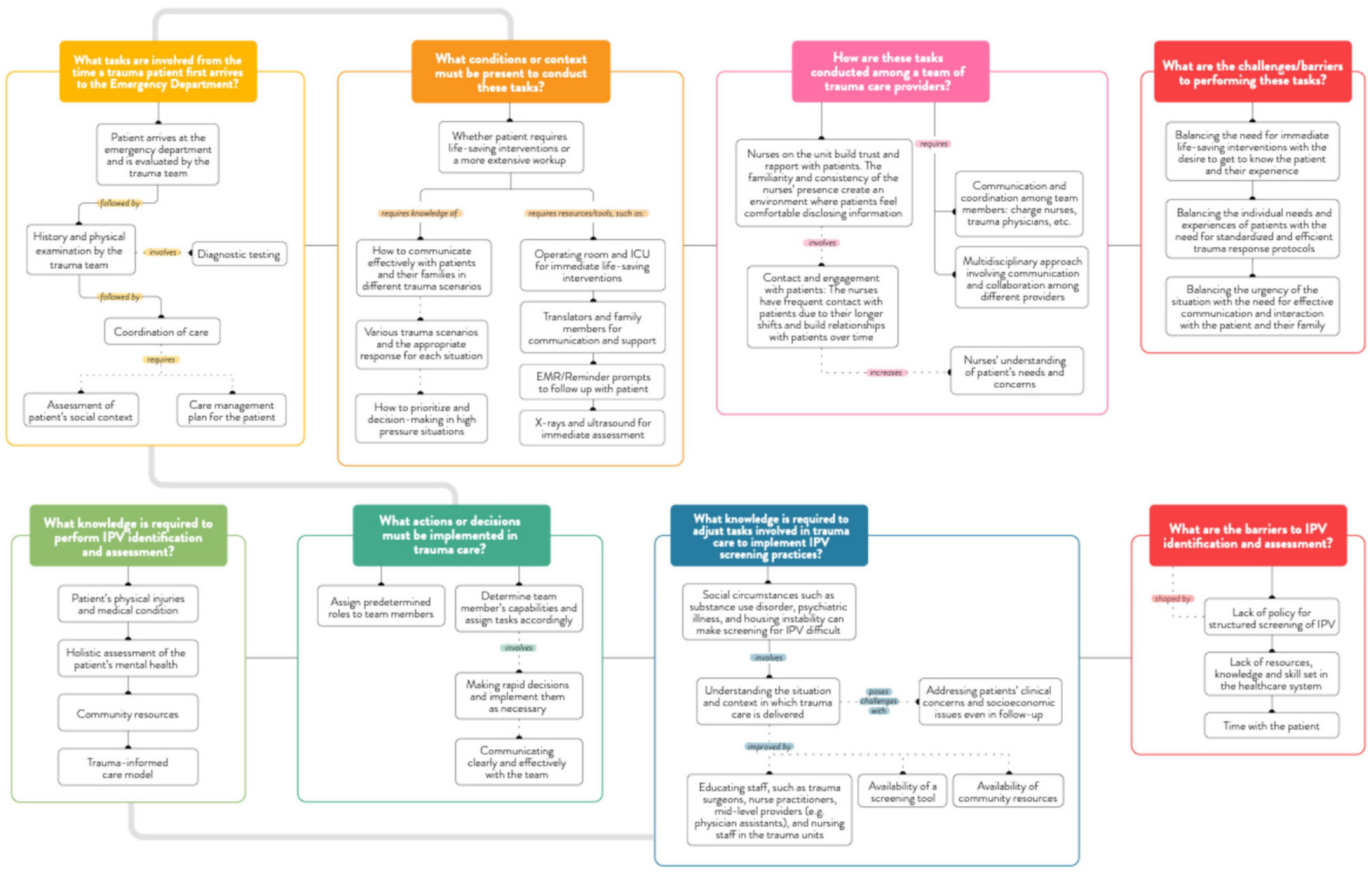
Cognitive processes of trauma care providers.

In the final **representing findings** phase, we synthesized insights into conceptual diagrams (Figures 2–7) and thematic narratives. These representations combined verbal data and concept maps to illustrate how IPV identification is cognitively and contextually integrated into trauma care tasks. The emerging themes depict both the real-time complexity of trauma care environments and the implicit decision strategies that influence providers' decisions on IPV identification and response. The concept map and themes were shared with participants for feedback, and the themes were refined during virtual meetings with the principal knowledge user of the study (NB).

### Ethical considerations

Ethical approval was obtained from the University of Alberta Research Ethics Board (Pro00119940). All participants were provided with information about the study and given the opportunity to give informed consent. Considering the group interview format, participants were reminded of the importance of maintaining confidentiality and engaging respectfully. Data were anonymized, securely stored, and only accessible to the research team.

## Results

Analysis of the group interviews revealed six main themes that reflected the macro-cognitive dimensions of trauma care providers' knowledge, experiences, and cognitive processes related to IPV identification and response. These themes included: (1) trauma care workflow, (2) team collaboration and knowledge, (3) critical situations and decision-making, (4) identifying IPV and challenges with screening, (5) understanding patient experiences with IPV, and (6) institutional support for IPV screening. Each theme is elaborated in detail below. Figure 7 illustrates a comprehensive concept map of trauma care providers' cognitive processes, structured by the open-ended, scenario-based questions that prompted participants to describe the real-life complexity of the trauma care environment.

In addition to the thematic findings described below, Table 2 provides a structured alignment of the identified barriers and facilitators with relevant CFIR domains/constructs and Proctor et al.'s implementation outcomes. This table does not represent a separate analytic phase or involve deductive coding; rather, it summarizes how empirically derived themes correspond to established implementation constructs, supporting transparency in how implementation-relevant insights were derived from the data.

**Table 2:** Alignment of identified barriers and facilitators with CFIR domains and proctor implementation outcomes.

Key finding (from CTA themes)	CFIR domain/construct	Proctor outcome(s) implicated	Illustrative example
High-acuity trauma workflow limits time for IPV inquiry	Inner Setting—Structural Characteristics; Available Resources	Feasibility	IPV screening deprioritized during resuscitation and early stabilization
Lack of standardized IPV protocol or EMR integration	Intervention Characteristics—Complexity; Compatibility	Feasibility; Appropriateness	Screening dependent on individual judgment rather than routine practice
Strong relational capacity among nurses and social workers	Characteristics of Individuals—Skills; Self-efficacy	Acceptability; Appropriateness	Trust-building enables delayed disclosure during admission
Unclear role responsibility for IPV follow-up	Inner Setting—Implementation Climate; Networks & Communications	Feasibility	Hesitation to screen due to uncertainty about “what happens next”
Concern about retraumatizing patients	Characteristics of Individuals—Beliefs about Consequences	Acceptability; Appropriateness	Providers delay or avoid screening without trauma-informed supports
Limited after-hours social work coverage	Inner Setting—Available Resources	Feasibility; Sustainability	IPV disclosures difficult to respond to during evenings/weekends

### Theme 1: trauma care workflow

Trauma care providers described a cognitively demanding workflow that begins with patient arrival in the ED and extends through to admission, stabilization, and interprofessional management (Figure 2). Providers emphasized the high acuity and rapid pace of trauma care, where the priority is to stabilize patients, perform diagnostic assessments, and implement life-saving interventions. The cognitive demands of this workflow were described as intense, requiring providers to synthesize clinical, social, and environmental information under time constraints. As one trauma nurse practitioner explained:

“Things happen pretty quickly, and it's like a bunch of people disseminating on a single individual, and things get done pretty fast. Our goal is always to have our resuscitation completed in under an hour.” (Trauma Nurse Practitioner)

Navigating this complex and critical environment means providers have to swiftly process clinical, social, and environmental factors with very little time. The urgency of trauma care means that immediate clinical priorities often dominate provider attention, leaving little space for broader psychosocial assessments—including those related to IPV. As one trauma surgeon emphasizes, “it's all about context.”

Participants noted that opportunities to consider IPV and other social determinants of health typically arise later in the care trajectory. However, there is no standardized process for IPV assessment, and the decision to screen or intervene is often left to the individual provider's judgment and availability of time and resources. A social worker described the layered and adaptive thinking required in such situations:

“…it's really important to be careful and not just jump into our own personal triggers or professional triggers or biases. And also, you don't always have time in a hospital setting, in an emergency department or even in ICU. You don't always have time to figure everything out. In that, I find it's really important to pay attention to, do I slow my thinking down or do I speed it up? And who else might be involved? I need to think, is there a child here? And how do I get in touch with this partner? What are the risks if he comes to the hospital? What's the level of intimate partner violence? Is the RCMP involved or EPS [referring to the local police service]? Is there a history of this? Is there a no contact order? There's just a lot of factors. I don't want to take up too much time. I think it's a good, concrete example that there are a lot of details that need to be attended to and sorted out as the professional.”

This quote reflects the dynamic decision-making processes involved in balancing clinical, safety, and contextual considerations in real time.

One trauma surgeon offered a nuanced perspective on why screening or assessing for IPV at the point of entry may not be appropriate, highlighting the physical and emotional vulnerability patients experience during trauma resuscitation:

“So, I think there's definitely a sense of vulnerability because the clothes are removed, and we cover them up with blankets and gowns and things, but there is definitely that vulnerability. Additionally, they may not necessarily know who is looking after them. And, I mean, we’ve always been advised to use universal precautions in trauma, but now, there's not even that. That connection with their providers, whether it's their nurses, residents, respiratory therapists, or attendings, is very different at the beginning when our goal is to stabilize them and assess for life-threatening injuries. That connection or any conversation with the patient is difficult when they’re lying horizontal with their neck [stabilized in a collar], and not able to even really move their head around.”

This quote highlights how the physical positioning, sensory disconnection, and depersonalization of acute trauma care can further disorient patients and inhibit trust-building, which is important for meaningful disclosure. In such moments, IPV inquiry might not only be ineffective but also potentially harmful or retraumatizing.

Several participants acknowledged that the biomedical focus of trauma care often sidelines psychosocial needs:

“The way the system is set up, the focus is on the physical, acute care health needs. And psychosocial concerns and addressing social determinants of health, it's painful to say, are secondary.”

Together, these reflections show how trauma care processes affect providers' cognitive demands and decision-making priorities. While the clinical environment permits some IPV-related inquiry, this often relies on individual initiative and informal judgment to identify when, where, and how to address IPV issues, highlighting the need for structural support to incorporate IPV screening programs into routine care (Figure 2).

### Theme 2: team collaboration and knowledge

Participants emphasized that trauma care is inherently team-based, requiring close collaboration among physicians, nurses, social workers, and other allied health professionals (Figure 3). Nurses were identified as key providers in building rapport with patients. They usually serve as the first point of trust with patients, which helps them notice signs of IPV and initiate supportive conversations. Social workers were similarly recognized for their specialized role in uncovering psychosocial risks and navigating the complexities of safety planning and resource navigation. One trauma surgeon expressed appreciation for the vital role nurses and social workers play in their trauma ward.

“A lot of times, our patients are in a state where it's very difficult to elicit any amount of even medical information, let alone social…And we come in the next day or in the afternoon, and we see our social workers knee-deep in all of this information that we just had no idea about because we were just focused on getting this patient stabilized and onto a unit or into an OR. Get them in, get them stable and get them out of the ED.”

In this interprofessional environment, the emotional intelligence and communication strategies used by team members—such as humour, empathy, and cultural humility—were described as essential to building patient trust. Interprofessional communication was viewed as both a strength and a challenge—while effective teams demonstrated seamless sharing of information, some reported uncertainty about who was responsible for initiating IPV screening and assessment. A conversation between two social workers highlighted the complex challenges of conducting IPV assessments in a hospital setting. One social worker described how disclosure is often delayed due to the pressures of the acute care environment:

“I think, in the acute care environment, there is a pressure, of course, to discharge people because of the bed demands. And these are really difficult conversations to have with people. They’re very sensitive subjects. And sometimes people do not disclose to us until near the end of their admission.”

Building on this reflection, another social worker emphasized the importance of relational trust and the compounding factors that shape patients' willingness to disclose:

“ But through the relationship development or relationship building that you’re having with that person, they do disclose. And then sometimes we are scrambling to, what do we do to help as much as we can? And there's also that cultural influence as well in terms of how often people disclose and how much they disclose, along with their personal experiences. […] And the hospital environment, it's clinical. It's very difficult for people sometimes to open up and have those conversations, especially if they’re trying to deal with their injuries and healing. Pain and just recovering from the injury itself could be a barrier.”

These reflections reveal that while strong interprofessional collaboration can enhance IPV detection and response, role clarity, the setting itself, and timing constraints often limit effective identification and response of IPV.

### Theme 3: critical situations and decision-making

Trauma care providers described critical clinical situations as cognitively demanding and highly structured, involving predefined team roles, rapid assessment, and coordinated task execution (Figure 4). In these moments, IPV screening was not prioritized unless physical signs or behavioural cues explicitly suggested abuse. Providers discussed the tension between their moral imperative to address psychosocial concerns and the clinical necessity of treating life-threatening injuries. A social worker shares the importance of predefined roles of providers within the trauma ward:

“As a system that moves fast and doesn't often have time to get into addressing social determinants of health and crises wholly and completely, we have to be wise, really wise with how we use the resources that we have, including ourselves as professional members of a multidisciplinary team…and I think that's also how we look after ourselves in these kinds of situations. Because I need nurses and doctors to be focused on the patient who needs certain kinds of medical intervention and care, and I also need to know what my role is and to do that. […] And also it's knowing when to ask questions and when not to. And who is the better person to do that?”

Leadership, situational awareness, and adaptive problem-solving were crucial cognitive skills employed during critical incidents. Some participants recounted situations where, after a patient had been stabilized, they intentionally revisited psychosocial concerns such as IPV, but these opportunities were inconsistently pursued.

### Theme 4: identifying IPV and challenges with screening

Participants acknowledged that formal IPV screening was uncommon and not systematically integrated into institutional protocols at both trauma centres. Although the electronic medical record (EMR) includes three to four safety-related questions, these are not derived from any validated or published IPV screening approach. Moreover, the EMR flowsheet containing these questions is accessible only to nursing staff and not to physicians, further limiting its use in routine trauma care. As a result, there was no standardized or consistently applied IPV screening tool embedded within the EMR system. Identification of IPV usually happens informally, triggered by obvious physical signs or injuries, patient behaviour, or collateral information shared by other staff. One social worker describes the cognitive and emotional considerations that shape IPV identification and assessment:

“it's thinking about right timing, risk assessment. What is involved in a safety plan? What's the motive? What's my self-reflection as a professional? And I might be motivated to help a person who is in an intimate partner violence situation more than they're motivated to help themselves. Or maybe it's not the right time. But we also know it takes many, many, many, many, many conversations, many, many, many attempts to leave before someone is actually able and ready.”

Additionally, a nurse shared a scenario where a patient's resistance to discharge prompted further inquiry. They shared an example of using informal team-based observations to validate suspicions:

“But when I asked the nurses are you seeing this kind of behaviour? This is what I’m picking up in my conversation with the patient, is that consistent with what they’re seeing? Because then I might actually reevaluate and go chitchat with the patient. I may do that on my own hunches, too, but often, I will go back. And again, because the nursing staff are the ones involved with the patient for longer periods of the day, they might have picked up another cue. Or the other day, they mentioned something interesting, and I’m, like, well, I wasn't aware of that. So, either that might confirm or not confirm my suspicion. But I look for collateral evidence before I circle back.”

Participants described multiple systemic and situational barriers that can hinder screening and identification practices (Figure 5). Time pressures, high patient volumes, a lack of standardized tools, limited provider knowledge, and inadequate institutional guidance and support were frequently described as challenges. One trauma surgeon reflected:

“I strongly believe that social circumstances like IPV require a far longer in-patient state or very close outpatient follow-up, regular outpatient visits, I think both to develop the therapeutic relationship and also it takes time to uncover and disclose a lot of these things. So, that's one of the barriers and I think that's the barrier of having a trauma service implement a screening tool and protocol. Because once you identify someone who's at risk, who does it fall upon to follow these patients? Who does it fall upon to ensure there's appropriate outpatient follow-up and check-in?”

Additionally, providers also expressed concern about retraumatizing patients or eliciting disclosures they were ill-equipped to manage. One nurse emphasized the importance of patient-centredness, particularly when screening processes are embedded in digital workflows:

“A screening tool is helpful in the sense that it makes you think about, this is something that we need to talk about, or potentially we need to talk about. But really, the patient is ultimately the one who guides that conversation. And the way that assessment is in Connect Care, it is very much a guide for the healthcare user. And we are the ones who are asking, but to me, it's not conducive to allowing the patient to lead the conversation and to speak when they want to speak.”

Participants noted that social workers were often relied upon to “pick up the pieces” in cases where IPV was disclosed, but this model was described as unsustainable. Furthermore, the intersection of IPV with other complex social issues—such as substance use, housing insecurity, and mental health—introduced added complexity and uncertainty around when and how to intervene. These overlapping issues contributed to hesitation and variability in IPV screening and response practices across trauma care providers.

### Theme 5: understanding patient experiences with IPV

Trauma care providers conveyed a strong desire to understand the lived experiences of patients affected by IPV (Figure 6). They recognized that building trust and rapport was foundational to eliciting disclosures and providing adequate care. Participants noted that patients may withhold information due to fear, shame, or cultural barriers, requiring providers to adopt a patient-centred and nonjudgmental approach. Some providers reported modifying their communication style—slowing down, sitting at eye level, using humour or small talk—to create a safer space for disclosure. The cognitive challenge of balancing empathy with clinical detachment was described as emotionally taxing but essential to trauma-informed practice. Others reflected on cases in which disclosure occurred only after repeated interactions, highlighting the importance of continuity and consistency in care. A trauma nurse practitioner recounted one example where continuity of medical care from a team was provided following a trauma patient disclosing IPV:

“Our medical perspective, if we have a patient who has disclosed IPV or some other violence, we’ve had patients who’ve had some pretty horrific sexual assaults as well, we try to have continuity in the medical team. Our medical team weekly consists of myself, a trauma surgeon, along with a rotating group of residents on service and off service, as well as medical students and NP students. In that case, we usually try to have one person who the patients build rapport with, who can provide continuity, be a real touch point.[…] And if there's any need to transition that person out, say, I’m going on two days off, we try to have a day where we overlap, and I would introduce the person who's going to be the next touch point for me. Often, in these situations, it is me because I provide the most continuity of care for our patients on the wards. And then I always like to make sure that they know, hey, I’m not going to be here tomorrow. I'm not going to be here for the next three days.”

### Theme 6: institutional support for implementing IPV screening programs

Participants across both trauma care sites consistently reported a lack of institutional protocols, policies and formalized training related to IPV screening and response. Providers described the current approach to IPV identification and assessment as reactive, fragmented, and highly variable across teams and shifts. Many participants expressed frustration with the absence of clear referral pathways, limited access to community-based resources, and insufficient administrative support. One trauma surgeon describes,

“So, I would say one of the barriers is, I guess, lack of leadership and resources, and disengagement of staff to perform their tasks. So, you identify someone who has IPV, then what? Well, we have no resources to offer, and we have nothing further, and we’re just putting you back to square one. So, I feel, from a systems perspective, we are failing these patients. And although not everything in life is about resources and money, this is a basic element of care that we'’re not able to provide patients consistently.”

Time constraints were frequently cited as a significant barrier to IPV screening or identification. Providers emphasized that screening for IPV requires more than a checklist—it can lead to disclosures that demand time, coordination, and follow-up. One trauma surgeon emphasized:

“I think one of the big barriers is going to be time. And the nurses on Unit 3F2 [referring to the specialized trauma and thoracic unit at the University of Alberta Hospital] are so overworked. Often, we don't have unit clerks, or we don't have health care aides, or sitters, or constants, I guess. And the amount of things that nurses are doing, so to screen for something like this, which it's not like have you had your COVID vaccines? This could, potentially, go down a rabbit hole that could take quite a while. So, I think the time that everybody has can definitely be one barrier.”

Staffing limitations—particularly the lack of dedicated social work coverage—further compounded these challenges. A patient care manager explains,

“I think a big barrier is our lack of social work support for trauma units. Actually, across the site, social work has been a huge challenge with staffing, and we only have social workers on Monday to Friday. A lot of times, patients are busy during the day with tests and rehab, and when they might want to talk is in the evenings, when they’ve had some downtime, and there's no one for them to talk to. Our staffing levels go down on evenings and nights, which is sometimes when families are available as well. And on weekends, we don't have any coverage for a social worker. So, that's a huge gap that could be filled by a consistent person who could have the time to spend with the patients.”

Training on IPV, when available, was optional and often limited to social work staff. Participants advocated for systemic solutions, including integrating standardized screening prompts into EHRs, mandating IPV training for all trauma care team members, and ensuring visible institutional leadership that prioritizes IPV as a serious clinical concern.

Several providers also emphasized the importance of aligning IPV screening with broader organizational commitments to trauma-informed care, cultural safety, and health equity. Without institutional buy-in and system-level supports, even well-intentioned efforts to identify IPV were likely to remain inconsistent and unsustainable.

In sum, the results of this study highlight a complex interplay of individual, interpersonal, and systemic factors that shape trauma care providers' ability to identify and respond to IPV. While there is recognition of the importance of IPV screening, substantial cognitive, emotional, and structural barriers inhibit its routine integration into trauma care workflows. These findings provide critical direction for designing and implementing contextually sensitive IPV interventions that are both feasible and sustainable in acute care settings.

## Discussion

This study provides new insights into the cognitive, organizational, and contextual factors that influence how trauma care providers identify and respond to IPV disclosures within acute care settings. Using CTA, we captured the tacit knowledge, reasoning strategies, and mental models that inform provider behaviour during real-time clinical decision-making. Our findings highlight key design considerations for IPV screening programs and improve understanding of the barriers to their implementation in trauma care, despite widespread recognition of their importance and the existence of evidence-based guidelines.

### Alignment of identified barriers and facilitators with CFIR domains and proctor implementation outcomes

Although CFIR and Proctor et al.'s implementation outcomes taxonomy were not used as deductive coding frameworks, they offered important conceptual lenses for understanding how cognitive, relational, and organizational factors influenced the feasibility of IPV screening in trauma care (see Table 2). Several CFIR domains/constructs and Proctor outcomes stood out across the findings. For example, at the CFIR inner setting level, structural factors (such as high-acuity workflows, staffing constraints, and limited social work coverage), implementation climate, and available resources determined whether IPV screening was seen as feasible or appropriate. Although this study was not designed to compare trauma centre levels, the inclusion of both a Level 1 and a Level 2 trauma centre provides important contextual insight into how organizational infrastructure shapes the cognitive and implementation conditions for IPV screening. Despite these structural differences, participants across the University of Alberta Hospital Trauma Centre (Level 1) and Royal Alexandra Hospital (Level 2) described similar cognitive pressures, workflow constraints, and reliance on informal judgment in identifying and responding to IPV. This suggests that the barriers identified in this study are not solely a function of resource availability, but are embedded in the core cognitive and organizational dynamics of acute trauma care. Among CFIR characteristics of individuals, providers' knowledge, beliefs about consequences, self-efficacy, and emotional labour were vital to screening decisions. Additionally, IPV screening was viewed as a complex intervention rather than a simple task, requiring time, trust, and coordination. The lack of integration with trauma workflows and EMR systems decreased perceived compatibility. Building on the alignment presented in Table 2, the discussion below focuses on what these implementation-relevant findings mean for the design and delivery of IPV screening programs in hospital trauma care.

### Bridging the gap between institutional policy and practice

Trauma care providers acknowledged the significance of IPV as a health and safety issue, yet the actual implementation of screening practices or identification of IPV was limited and inconsistent. This disconnection between institutional policy on IPV screening and identification and day-to-day practice reflects broader challenges in integrating psychosocial assessments into clinical care environments. Providers highlighted numerous cognitive and contextual barriers to screening and identification of IPV, including competing clinical priorities, a lack of clarity around professional roles, emotional labour associated with IPV disclosures, time constraints, and insufficient institutional resources. These findings are consistent with prior research documenting the underutilization of IPV screening in emergency and trauma care (14, 35).

Our study expands on this body of work by revealing how these structural and practice barriers manifest cognitively. For example, trauma care providers described the emotional and mental effort required to switch between life-saving interventions and the relational work needed to encourage and support IPV disclosures. These shifts are not merely logistical—they reflect different ways of thinking, requiring providers to transition from algorithmic decision-making to complex judgment and emotional sensitivity.

### The role of cognitive demands and mental models

Using CTA allowed us to explore the mental models that guide trauma providers' IPV-related judgments. Participants' conceptions of *when* and *how* IPV assessment becomes relevant often depended on observable injuries or patient disclosures, rather than a proactive or standardized approach. While providers demonstrated strong situational awareness and team-based coordination during high-acuity care, they lacked a shared cognitive framework for integrating IPV screening or identification into routine assessments. This fragmentation resulted in *ad hoc* approaches, typically initiated by providers with greater confidence or perceived role alignment, such as nurses or social workers.

These findings highlight the need to rethink how IPV is understood within trauma care. Instead of viewing IPV as a secondary concern, it should be regarded as central to patient safety and recovery. This shift requires both cognitive and structural changes, including creating shared mental models, normalizing screening practices, and integrating IPV-related cues into diagnostic reasoning and decision-making pathways.

### Relational work as core to trauma-informed IPV care

The study underscored the critical role of relational work in facilitating IPV disclosure. Nurses, in particular, emerged as relational gatekeepers—using empathy, humour, and continuity to build trust with admitted patients. However, this relational labour is often invisible, unrecognized in formal workflows, and unsupported by institutional systems. Moreover, providers expressed concern about the emotional toll of repeated IPV encounters without appropriate training, debriefing, or systemic follow-up. These findings align with trauma-informed care literature, which emphasizes the need for institutional cultures that recognize and support the relational aspects of healing (36).

### Factors informing the design of IPV screening programs

Findings from the CTA provide important insights into how trauma care providers cognitively navigate the complex and time-sensitive environment of acute care, offering guidance for how IPV screening programs can be designed and integrated. First, the findings emphasize that the *timing* of IPV screening must be aligned with the trauma care trajectory. Immediate screening during resuscitation or early stabilization is not appropriate, as patients are physically vulnerable, cognitively overwhelmed, and unfamiliar with providers. Instead, screening efforts should be initiated once clinical stabilization is achieved, have had time to orient to their surroundings, and a therapeutic relationship has begun to form—typically later in admission or during early recovery. Second, the findings suggest that *who* conducts IPV screening and assessment matters. While social workers are often relied upon, a more distributed, team-based model of responsibility is needed (22). Nurses and allied health providers who spend prolonged time with patients are well-positioned to detect subtle cues and follow up on suspicions and disclosures collaboratively.

### Conditions influencing the implementation of IPV screening programs

Trauma care team members require clear roles, adequate training, and support to navigate disclosures effectively. Moreover, the effectiveness of screening is contingent not only on who asks and when, but also on how it is conducted—requiring trauma-informed, patient-led approaches grounded in trust and cultural safety. Complexity science highlights the importance of emergent factors, such as provider workload, relational dynamics, and patient readiness, which can vary over time and across contexts.

### Implementation strategies for IPV screening

Comprehensive IPV screening and response efforts must be systemically supported through formal protocols, consistent staff training, embedded screening tools in the EHR, and sufficient staffing—particularly access to social work beyond weekday daytime hours. Without these institutional supports, screening remains informal and reliant on individual judgment, limiting its consistency and impact. These strategies support the implementation of contextually sensitive IPV screening interventions that are feasible within the cognitive and operational realities of trauma care.

Mapping findings to CFIR and Proctor's taxonomy also clarifies where implementation strategies should be targeted. To improve feasibility (Proctor), system-level strategies that address CFIR inner setting constructs are necessary, including expanded social work coverage, clear referral pathways, and EMR-integrated prompts aligned with trauma workflows. Enhancing appropriateness involves intervention adaptability, allowing IPV screening to occur later in admission when cognitive load is reduced and relational trust has been established. Improving acceptability depends on investing in provider training that addresses emotional labour, trauma-informed communication, and shared mental models across disciplines. Importantly, our CTA findings highlight that implementation strategies must reduce, not add to, cognitive burden. Embedding comprehensive IPV screening practices within existing team roles, decision points, and documentation systems is more likely to support sustained uptake than introducing standalone screening interventions.

### Advancing implementation and complexity science

This study contributes to implementation science by deepening the understanding of how cognitive and contextual factors influence the feasibility of IPV screening interventions in complex clinical settings. Using principles of complexity science, our CTA approach demonstrated that IPV screening and assessment is not a linear process, but an adaptive practice embedded within a dynamic system marked by fluctuating acuity, shared cognitive work across the trauma care team, variable team composition, and significant emotional labour. Instead of focusing solely on the adoption of a screening protocol, our findings emphasize the importance of designing flexible, system-aware implementation strategies that align with the real-time workflow, mental models, and tacit decision-making processes of trauma care providers. The study highlights key points for future implementation research on IPV screening interventions, such as embedded decision supports, iterative training based on real-world practice, and adaptive feedback mechanisms that facilitate ongoing learning and adjustment. Incorporating complexity-informed strategies—such as sensemaking, distributed leadership, and context-specific facilitation—may better support the adoption, sustainability, and fidelity of IPV screening programs in high-pressure care environments like hospital trauma care centres. These findings further emphasize the need for research on relational implementation strategies (37) informed by complexity, focusing not only on context and process but also on providers' dynamic mental capacity as they navigate high-stakes care environments and the structural vulnerabilities that shape patient experiences in trauma care.

### Strengths and limitations

A strength of this study is its use of CTA to uncover the cognitive dimensions of IPV identification and screening, offering a level of depth and specificity rarely captured in standard qualitative interviews. Our interprofessional participant sample and concept mapping approach added further richness to the data. Additionally, this research brings implementation science closer to the *cognitive realities of clinical practice*. While implementation frameworks often consider contextual, organizational, and behavioural factors, they rarely delve deeply into the real-time cognitive strategies that influence provider behaviour—such as situational awareness, mental load, implicit decision rules, and adaptive judgment (38–40). By using CTA, we identified how trauma providers perceive, prioritize, and respond to IPV-related cues amidst competing demands. This adds nuance to our understanding of contextual factors and highlights the need for implementation strategies that account for cognitive demand, implicit mental models, and clinical judgment—not just attitudes or knowledge deficits.

However, the study also has limitations. The sample size was small and limited to two hospitals within a single urban region, potentially limiting generalizability. Group interview dynamics may have influenced what participants shared, and the absence of patient perspectives is a notable gap.

## Conclusion

This study advances understanding of the cognitive and contextual factors that shape provider decision-making and highlights the challenges of implementing IPV screening programs in acute care environments such as trauma care. By surfacing the tacit knowledge and cognitive challenges embedded in trauma care, these findings offer actionable insights for designing trauma-informed, context-sensitive IPV screening programs that are responsive to real-world constraints. As health systems increasingly commit to equity and safety, embedding IPV screening and identification in hospital trauma care advances the United Nations' Sustainable Development Goals (SDG) #3 (Good Health and Well-Being) and #17 (Partnerships for the Goals) by improving population health outcomes and well-being through system-wide collaboration and integrated care.

## Data Availability

The raw data supporting the conclusions of this article will be made available by the authors, without undue reservation.

## Appendix A

***Understanding cognitive aspects of the tasks involved in trauma care***

Can you describe the tasks (including workflow and procedures) that are involved from the time of a trauma patient's first arrival to the Emergency Department (e.g., trauma assessment) and admission to trauma service (or non-trauma service if trauma is stabilized)?
What conditions or context must be present to conduct these tasks?
What are the reasons for these tasks? How are these tasks conducted among a team of trauma care providers?
What knowledge is required to perform these tasks? (Probe: what do you have to know or what information is needed to make critical decisions regarding trauma care)
What actions and/or decisions must be implemented?

***Implementing IPV screening practices in trauma care***

How is IPV among in-patient trauma patients currently identified and assessed in hospital?
What do you perceive as the barriers to IPV identification and assessment among trauma in-patients? Can you describe any barriers or elements within your tasks that would prevent you from screening IPV?
Can you think of a time when you had cared for a trauma patient who had sustained an IPV-related injured? Was IPV disclosed or suspected?
Can you think of a time when you had improvised a task(s) to assess whether a patient was exposed to IPV?
Hypothetically speaking, within the tasks described earlier (workflow, procedures, practices) when would you screen or assess for IPV? Can you describe the setting/context in which this would occur?
Can you describe an instance where you had to change the way you normally performed a task(s) (or in other words your job) to support a patient who had self-disclosed IPV?
What knowledge and processes are required to adjust the tasks involved in trauma care to implement IPV screening practices?
What resources or institutional supports are required to perform IPV screening within your practice? What institutional changes are needed to implement IPV screening practices?
What performance standards must be achieved? (Probe: training and education, quality improvement)
